# Combined pulmonary fibrosis and emphysema: a retrospective analysis of clinical characteristics, treatment and prognosis

**DOI:** 10.1186/s12890-016-0300-7

**Published:** 2016-11-03

**Authors:** Lijuan Zhang, Chunling Zhang, Fushi Dong, Qi Song, Fangzhou Chi, Lu Liu, Yupeng Wang, Chunli Che

**Affiliations:** 1 Department of Respiratory Medicine, First Affiliated Hospital of Harbin Medical University, 23 Youzheng Avenue, Nangang District, Harbin, Heilongjiang China; 2 Department of Clinical Medicine, Harbin Medical University, Harbin, Heilongjiang China; 3 Department of Health Statistics, Harbin Medical University, Harbin, Heilongjiang China

**Keywords:** Combined pulmonary fibrosis and emphysema, Idiopathic pulmonary fibrosis, The composite physiologic index, Computed tomography

## Abstract

**Background:**

Combined pulmonary fibrosis and emphysema (CPFE) is increasingly acknowledged as a separate syndrome with distinct clinical, physiological and radiological characteristics. We sought to identify physiologic and radiographic indices that predict mortality in CPFE.

**Methods:**

Data on clinical characteristics, pulmonary function, high-resolution computed tomography (HRCT) and treatment were compared between patients with usual interstitial pneumonia (UIP) plus emphysema (CPFE group) and those with IPF alone (IPF group). Composite physiologic index (CPI) and HRCT scores at diagnosis and during follow-up were assessed.

**Results:**

CPFE group (*N* = 87) was characterized by the predominance of males and smokers, who were less likely to have viral infection prior to the diagnosis, and display basal crackles, finger clubbing and wheeze, as compared to that in the IPF group (*N* = 105). HRCT and CPI scores increased over time in both groups. Moreover, CPFE group had a poorer prognosis, lower 5-year survival rate (43.42 % *vs*. 65.56 %; *P* < 0.05), and higher mortality (39.47 % *vs*. 23.33 %; *P* < 0.05) as compared to that in the IPF group. All CPFE patients received oxygen therapy, antibiotics and oral N-acetylcysteine; > 50 % received bronchodilators, 40 % received corticosteroids and 14 % needed noninvasive mechanical ventilation. On survival analyses, pulmonary arterial hypertension (PAH) and ≥ 5-point increase in CPI score per year were predictors of mortality in the CPFE group (hazard ratio [HR]: 10.29, 95 % Confidence Interval [CI]: 2.69–39.42 and HR: 21.60, 95 % CI: 7.28–64.16, respectively).

**Conclusion:**

Patients with CPFE were predominantly male and smokers and exhibited distinct clinical, physiological and radiographic characteristics. They had a poorer prognosis than IPF. PAH and ≥ 5-point increase in CPI score per year were predictors of mortality in these patients. Future studies are needed to identify the optimal treatment approach to CPFE.

## Background

Patients with idiopathic pulmonary fibrosis (IPF) combined with emphysema were first reported several decades ago [1–3]. In 1990, Wiggins et al. conducted a retrospective study of eight patients who had high-resolution computed tomography (HRCT) findings of co-existing cryptogenic fibrosing alveolitis and emphysema [4]. Recent studies have indicated a more frequent association of emphysema with IPF than was previously thought. This study led to the characterization of the syndrome of combined pulmonary fibrosis and emphysema (CPFE), based on radiographic findings of upper lobe emphysema, and lower lobe pulmonary fibrosis (PF). Among the interstitial lung diseases (ILD) with concomitant emphysema, IPF is the most frequent.

In clinical practice, CPFE cases are not uncommon, and the estimated prevalence of emphysema is thought to range between one quarter and one half of all IPF patients [5, 6]. In the past 10 years, this syndrome has increasingly been acknowledged as a separate clinical entity. The syndrome is characterised by distinct symptomatology, clinical manifestations, radiological and histopathological features, and prognosis [7, 8]. CPFE is most often observed in males with a mean age of 65–70 years [7, 9–12]. Clinical features include severe dyspnea on exertion, subnormal spirometry findings, severely impaired gas exchange, hypoxemia on exercise, and characteristic findings on imaging [9, 11, 12]. More importantly, CPFE has a dismal prognosis with a 5-year survival rate of 55 % [9]. CPFE is associated with higher mortality and lower median survival time (25 months) as compared to IPF (34 months) [6]. Pulmonary arterial hypertension (PAH) is known to develop in up to half of all patients with CPFE, and has been identified as a key determinant of prognosis [6, 9]. A few studies have suggested pulmonary function parameters and the composite physiologic index (CPI) as being predictors of CPFE prognosis [5]. However, the prognostic value of quantitative physiologic and imaging characteristics has not been adequately investigated. Finally, the therapeutic options for patients with CPFE are limited, and there are no consensus recommendations for treatment of emphysema in the setting of IPF.

In this study, we retrospectively compared the clinical and radiographic characteristics, available therapeutic options, and prognostic indicators between patients with CPFE and those with IPF alone. Furthermore, we assessed the prognostic significance of quantitative physiologic and radiographic indices in patients with CPFE.

## Methods

### Subjects

Subjects were genetically unrelated ethnic Northern Han Chinese and from Heilongjiang and Harbin Province. All hospitalized patients were consecutively recruited from January 2001 to December 2013 in the Department of Respiratory Medicine at the First Affiliated Hospital of Harbin Medical University. We included two groups: patients with CPFE who had usual interstitial pneumonia (UIP) pattern according to HRCT, and patients with IPF alone. The patients in the CPFE group met the diagnostic criteria for IPF for patients not subjected to surgical lung biopsy (in accordance with the 2011 American Thoracic Society and European Respiratory Society (ATS/ERS) statement) [13]. Additionally, they had a chest HRCT scan showing co-existing emphysema and PF, well-demarcated areas of decreased attenuation delineated by a very thin (<1 mm) or no wall, multiple bullae (>1 cm) with upper zone predominance, reticular abnormalities with subpleural and basal predominance, honeycombing, architectural distortion, traction bronchiectasis, and/or ground-glass opacities, and/or areas of alveolar consolidation [9]. Patients diagnosed with conditions that are known to cause PF such as drug-induced interstitial lung disease, pneumoconiosis, hypersensitivity pneumonitis, sarcoidosis, pulmonary histiocytosis, lymphangioleiomyomatosis and eosinophilic pneumonia, were excluded from the study. In addition, PAH describes a subpopulation of patients with Pulmonary hypertension (PH) characterized hemodynamically by the presence of pre-capillary PH including an end-expiratory pulmonary artery wedge pressure (PAWP) ≤ 15 mmHg and a pulmonary vascular resistance > 3 Wood units [14].

### Methods

Medical records, pulmonary function tests and HRCT scans at diagnosis and during the 60-month follow-up period, were analyzed. The composite physiologic index (CPI) that represents a combination of pulmonary ventilation and diffusing capacity, and chest HRCT score were obtained to evaluate the extent of disease at diagnosis and at 6 months intervals up to a total of 36 months. The formula for the CPI is as follows: CPI = 91.0−(0.65 × percent predicted diffusing capacity for carbon monoxide-DLCO])−(0.53 × percent predicted forced vital capacity [FVC]) + (0.34 × percentage of predicted forced expiratory volume in 1 s [FEV1]) [15].

HRCT scans were evaluated separately by two radiologists using an image-processing program (ImageJ, version Windows 32-bit 1.6.0_24, a public program available at (https://imagej.nih.gov/ij/download.html). The extent of emphysema was visually estimated bilaterally in HRCT sections at three anatomic levels: near the superior margin of the aortic arch (the upper lung field), the carina (the middle lung field), and at 1 cm above the right hemidiaphragm (the inferior lung field). A score was assigned to grade the emphysematous changes in each lung field as follows: score 0 (no emphysema), score 0.5 (trivial, < 5 %), score 1 (mild, 5–25 %), score 2 (moderate, 26–50 %), score 3 (marked, 51–75 %), and score 4 (severe, > 75 %). The severity of emphysema was graded based on the sum of the scores in all six lung fields as follows: score 0 (no emphysema), score 1–8 (mild); score 9–16 (moderate), score 17–24 (severe) [16]. PF was assessed in HRCT sections at three anatomic levels. The extent of fibrosis was quantified as percent lung parenchyma affected at each section, as well as the cumulative involved lung by ground glass opacities, interlobular septal thickening, reticular opacities, and honeycombing, respectively, according to the following scoring system: score 0 (not affected), score 1 (≤5 %), score 2 (6–24 %), score 3 (25–49 %), score 4 (50–74 %), score 5 (≥75 %) [17]. PF score was adjusted by multiplying the coefficient 4.8, which allowed the severity of fibrosis to be graded similar to that of emphysema [18]. The final HRCT score for CPFE was the sum of emphysema score and fibrosis score.

The study endpoint was the completion of the 60-month follow-up, including all-cause deaths and loss to follow-up. Mortality of CPFE was calculated from the deaths that directly resulted from CPFE or CPFE-related causes.

### Statistical analysis

Continuous variables are presented as mean ± standard deviation (SD); intergroup differences were assessed by performing *t*-test. Intragroup variability was assessed by repeated Analysis of Variance (ANOVA). Categorical variables are presented as percentages and compared using the chi-square test and Fisher’s exact test. Univariate and multivariate survival analyses were performed to determine the relationship between clinical parameters, physiological indices, HRCT imaging scores and survival. Differences with an associated *P* value of < 0.05 were considered significant. All statistical analyses were performed using SAS version 9.1.3 software.

## Results

### Clinical characteristics and outcomes

A total of 87 patients were included in the CPFE group (79 males; 69 ± 8.5 years). The IPF group included 105 patients (66 males; 60 ± 4.3 years). The demographic, clinical characteristics and outcomes in both the groups are presented in Table 1.

**Table 1:** Demographic variables, clinical characteristics and outcomes by study group

Group	CPFE (*N* = 87	IPF (*N* = 105)	Chi-square value	*P*
Age, years	66 ± 8.5	60 ± 4.3	0.63	<0.0001^*^
Male	76	66	14.83	0.0001
BMI < 18 kg/m^2^	37	39	0.58	0.4475
Smokers	75	44	39.63	<0.0001
History of viral infection	13	37	10.18	0.0014
Diabetes	16	10	3.19	0.0739
Cardiovascular and cerebrovascular diseases	67	63	6.29	0.0121
Cancer	8	8	0.15	0.694
Cor pulmonale	21	25	0.01	0.9577
PAH	39	45	0.08	0.7841
Finger clubbing	13	11	0.87	0.3516
Wheeze	55	12	56.17	<0.0001
Basal crackles	47	97	37.34	<0.0001
CEA increasing	21	6	49.11	<0.0001
5-year survival, %	43.42 (33/76)	65.56 (59/90)	9.37	0.023
All-cause mortality, %	56.58 (43/76)	34.44 (31/90)	14.33	<0.001
Direct-cause mortality, %	39.47 (30/76)	23.33 (21/90)	22.14	<0.001

Data presented as *N* (%) or mean ± standard deviation (range); Chi-square test was performed for assessing statistical significance, unless indicated otherwise

*Abbreviations*: *CEA* carcinoembryonic antigen, *PAH* pulmonary arterial hypertension

^*^Age compared by *t*-test

The mean age at diagnosis, and the proportion of males and smokers in the CPFE was higher than that in the IPF (*P* < 0.05 for all). Patients with CPFE had less frequent history of viral infection as compared to that in the IPF group (*P* < 0.05). CPFE patients were more likely to have concomitant cardiovascular and cerebrovascular diseases than IPF patients (*P* < 0.05). CPFE patients more frequently presented with wheeze (63.2 % *vs*. 11.4 %, respectively), and less frequently with basal crackles, than IPF patients (*P* < 0.05 for both). These were more likely to show concomitant cardiovascular and cerebrovascular disease, wheeze (63.2 % *vs*. 11.4 %), and an increased level of carcinoembryonic antigen (CEA) as compared to patients in the IPF group (*P* < 0.05). There was no significant difference in the proportion of patients with BMI < 18 kg/m^2^ between the two groups (*P* > 0.05). At the completion of the 60-month follow-up period, 33 patients with CPFE were alive; 11 were lost to follow-up; 30 died from CPFE or CPFE-related causes (lung cancer excluded); 11 died from extrapulmonary diseases, and 2 died from lung cancer. The mean 5-year survival rate in the CPFE group was 43.42 %, with an all-cause mortality rate of 56.58 %, and CPFE-related mortality of 39.47 %. By comparison, 59 IPF patients were alive at the end of 60 months, 15 were lost to follow-up; 21 died from IPF or IPF-related causes, 18 from extrapulmonary diseases and 2 from lung cancer. The mean 5-year survival rate of IPF patients was 65.56 %, with a mean all-cause mortality rate of 34.44 %, and IPF-related mortality of 23.33 %. Compared to IPF, CPFE had a significantly lower 5-year survival rate, higher all-cause mortality and disease-related mortality (*P* < 0.05).

### Longitudinal analyses of quantitative physiologic and radiographic

**Fig. 1:**
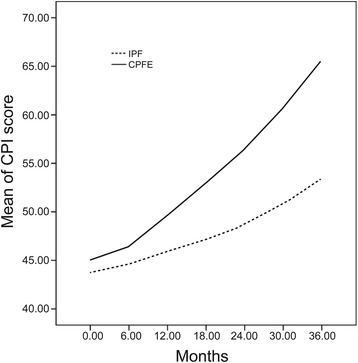
Longitudinal trend of mean CPI scores by study group during follow-up. CPI, Composite physiologic index; IPF, Idiopathic pulmonary fibrosis; CPFE, Combined pulmonary fibrosis and emphysema

**Fig. 2:**
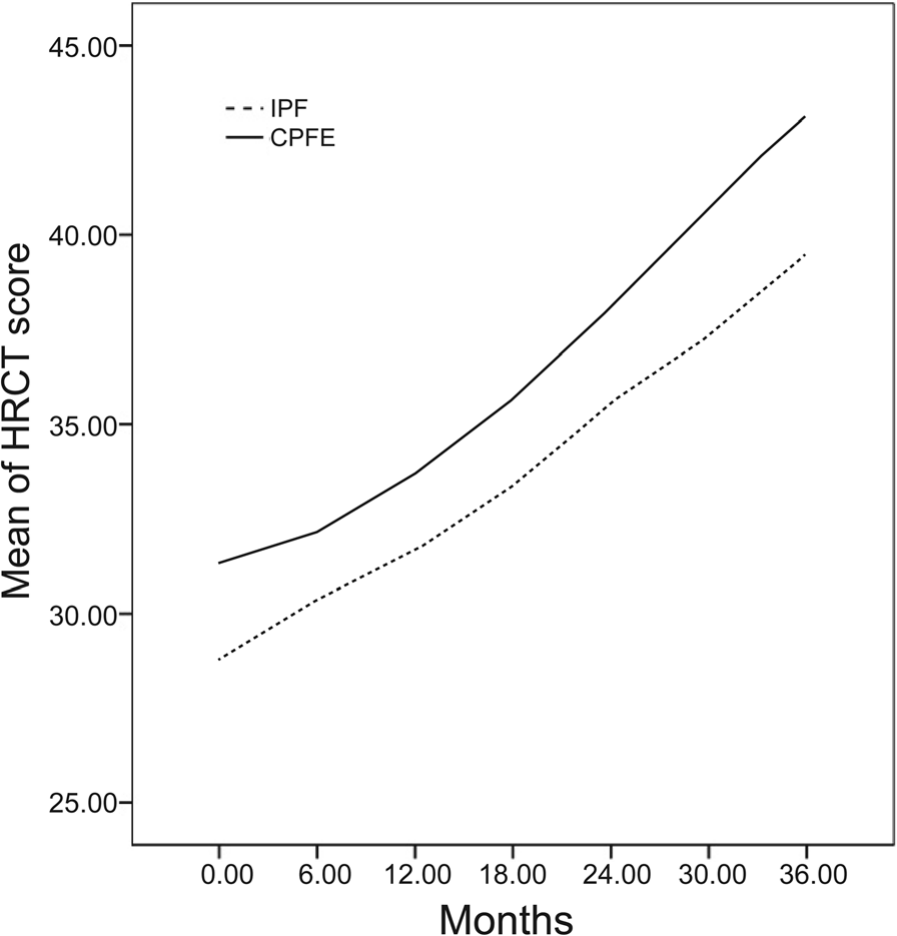
Longitudinal trends of mean HRCT scores by study group. HRCT, High-resolution computed tomography; IPF, Idiopathic pulmonary fibrosis; CPFE, Combined pulmonary fibrosis and emphysema

The CPI and HRCT scores were used to evaluate the disease progression in both groups and were recorded at 6-month intervals for a total duration of 36 months. During follow-up, all-cause deaths were included in the analysis, whereas data pertaining to patients who were lost to follow-up were excluded. A total of 80 CPFE and 99 IPF completed the follow-up with complete data available for analyses (Tables 2 and 3; Figs. 1 and 2). The CPIs of patients with CPFE were significantly higher than those of patients with IPF at all time points during the course of the disease (*P* < 0.05) (Table 2). As shown in Fig. 1, the CPI score of the CPFE increased more dramatically as compared to that in the IPF group over the 36 months of follow-up, which suggests a more rapidly progressive clinical course of CPFE as compared to that of IPF. The same tendency was observed with respect to HRCT scores (*P* < 0.05).

**Table 2:** Mean CPI scores by study group during follow-up

Group	0 month	6 months	12 months	18 months	24 months	30 months	36 months
IPF	43.76 ± 8.74	44.68 ± 8.67	45.93 ± 8.58	47.14 ± 8.53	48.67 ± 8.5	50.78 ± 8.49	53.36 ± 8.48
CPFE^*^	45.04 ± 8.17	46.45 ± 7.78	49.56 ± 7.91	52.96 ± 8.19	56.4 ± 9.22	60.6 ± 9.51	65.55 ± 10.26

Data are presented as mean ± Standard Deviation

*Abbreviations*: *CPI* composite physiologic index, *IPF* idiopathic pulmonary fibrosis, *CPFE* combined pulmonary fibrosis and emphysema

^*^ *P* < 0.05 compared to the changes in IPF group

**Table 3:** Mean HRCT scores by study group during follow-up

Group	0 month	6 months	12 months	18 months	24 months	30 months	36 months
IPF	28.79 ± 3.12	30.34 ± 3.02	31.67 ± 3.01	33.36 ± 2.83	35.51 ± 3.04	37.35 ± 3.15	39.46 ± 2.88
CPFE^*^	31.32 ± 5.07	32.16 ± 5.08	33.7 ± 5.05	35.68 ± 5.13	38.09 ± 5.41	40.7 ± 5.25	43.21 ± 4.68

Data are presented as mean ± Standard Deviation

*Abbreviations*: *HRCT* high-resolution computed tomography, *IPF* idiopathic pulmonary fibrosis, *CPFE* combined pulmonary fibrosis and emphysema

^*^ *P* < 0.05 compared to the changes in IPF group

### Treatment

**Fig. 3:**
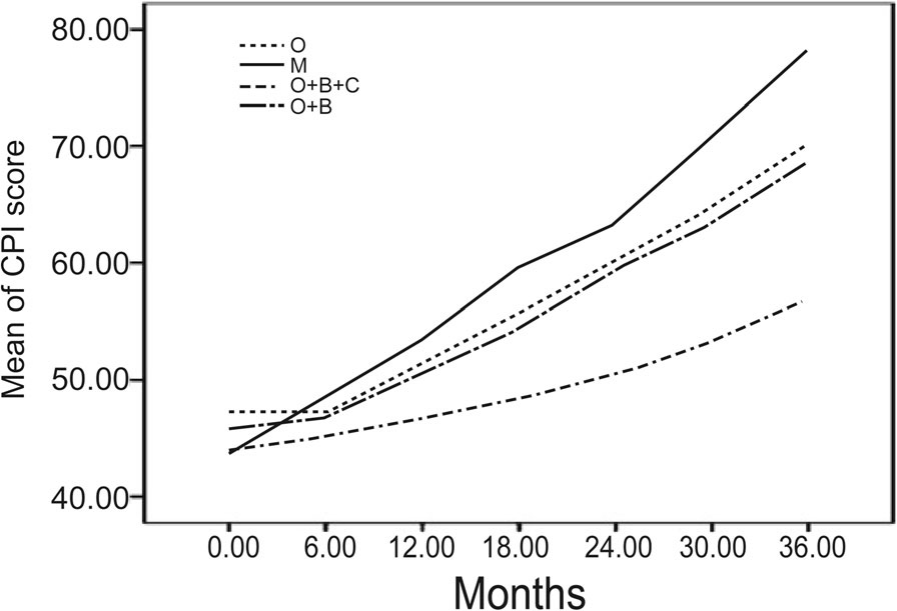
Longitudinal trends of mean CPI scores in CPFE patients disaggregated by therapeutic regimen. CPI, Composite physiologic index; CPFE, Combined pulmonary fibrosis and emphysema; O: Oxygen therapy; M: mechanical ventilation; B: bronchodilators; C: corticosteroids

**Fig. 4:**
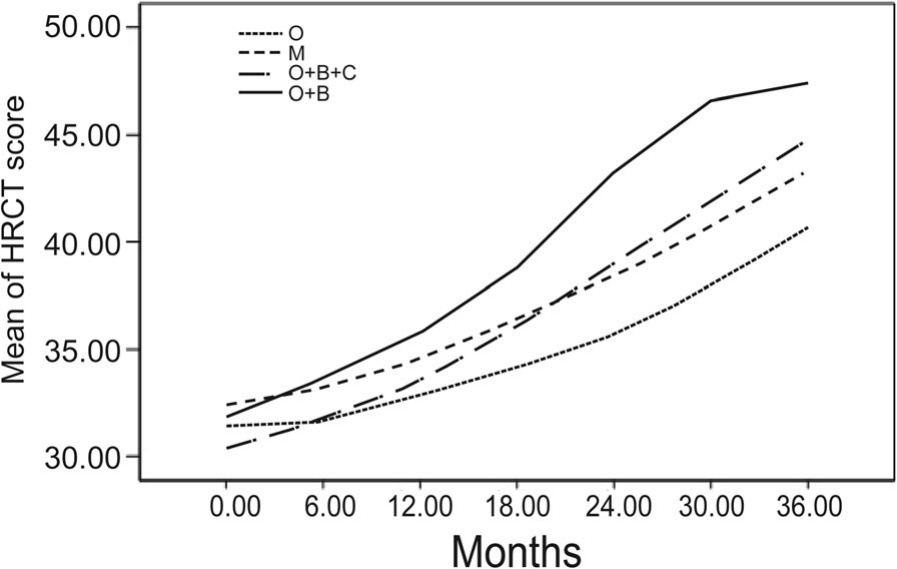
Longitudinal trend of mean chest HRCT scores in CPFE patients disaggregated by therapeutic regimen. HRCT, High-resolution computed tomography; CPFE, Combined pulmonary fibrosis and emphysema

The effect of various therapeutic regimens on prognosis of CPFE patients were assessed by CPI and HRCT scores in sub-groups of patients receiving different treatment. All 87 patients with CPFE received oxygen therapy, antibiotics and oral N-acetylcysteine treatment. Out of these, 12 patients who experienced acute exacerbation or respiratory failure during the disease course were administered noninvasive mechanical ventilatory support; 36 received bronchodilators plus corticosteroids and 15 received bronchodilators but no corticosteroids. The treatment lasted for at least 2 months per year. Rare cases that received other treatments such as immunosuppressants and anti-fibrotic targeted therapy were excluded from the analyses. At the end of the 36-month follow-up, complete data on the CPIs and HRCT scores were available for 69 patients in the CPFE group. These were divided into four sub-groups based on the treatment regimen (Tables 4 and 5). As shown in Table 4, significant differences were observed with respect to the CPIs in the different treatment groups at each time point of follow-up (F = 9.73; *P* < 0.0001). The rate of increase in CPI scores showed significant differences between the four different treatment groups, as well as the interaction effects of different time points during follow-up (F = 24.31; *P* < 0.0001). The rate of increase in CPI score in the group treated with oxygen therapy, bronchodilators and corticosteroids was lower than that in the other groups, which suggests that this therapeutic regimen may be more effective, especially if administered within 24 months from diagnosis (Fig. 3). In contrast, patients on mechanical ventilation support deteriorated dramatically with an abrupt increase in CPI score. Significant differences in HRCT scores were also observed among the different treatment groups at each time point of follow-up, as well as with respect to the interaction effects at different time points (F = 3.3882, *P* = 0.02182; F = 14.9756, *P* < 0.0001). The trend of HRCT and CPI among different treatment groups is shown in Fig. 4.

**Table 4:** Mean CPI scores in CPFE group disaggregated by therapeutic regimen on follow-up

Group	0 month	6 months	12 months	18 months	24 months	30 months	36 months
O	47.23 ± 7.98	47.24 ± 7.4	51.35 ± 7.55	55.68 ± 6.19	60.06 ± 6.28	64.86 ± 6.24	70.2 ± 6.39
M	43.55 ± 8.82	48.5 ± 7.79	53.41 ± 7.35	59.72 ± 7.41	63.22 ± 11.99	70.83 ± 7.87	78.22 ± 7.22
O + B + C^*^	43.79 ± 8.08	45.1 ± 7.83	46.65 ± 7.36	48.32 ± 7.52	50.45 ± 7.12	53.19 ± 6.96	56.96 ± 6.88
O + B	45.71 ± 8.14	46.77 ± 8.45	50.62 ± 8.48	54.37 ± 7.46	59.36 ± 7.35	63.36 ± 7.21	68.59 ± 6.76

Data presented as mean ± standard deviation

*Abbreviations*: *CPI* composite physiologic index, *CPFE* combined pulmonary fibrosis and emphysema, *O* oxygen therapy, *M* mechanical ventilation, *B* bronchodilators, *C* corticosteroids

^*^ *P* < 0.05 compared to changes in O group

**Table 5:** Mean chest HRCT scores in CPFE patients disaggregated by therapeutic regimen on follow-up

Group	0 month	6 months	12 months	18 months	24 months	30 months	36 months
O	31.33 ± 4.62	31.61 ± 4.47	32.83 ± 4.69	34.11 ± 4.85	35.69 ± 5.01	37.92 ± 4.94	40.67 ± 5.08
M	32.4 ± 3.92	33.13 ± 4.49	34.53 ± 4.53	36.33 ± 4.7	38.4 ± 4.75	40.67 ± 4.37	43.33 ± 3.22
O + B + C^*^	30.38 ± 6.02	31.67 ± 6.12	33.46 ± 5.93	36.04 ± 5.76	38.96 ± 5.69	41.96 ± 4.65	44.83 ± 3.71
O + B	31.83 ± 5.84	33.58 ± 5.42	35.75 ± 4.59	38.83 ± 3.64	43.17 ± 2.08	46.58 ± 1.16	47.42 ± 0.79

Data presented as mean ± SD

*Abbreviations*: *HRCT* high-resolution computed tomography, *O* oxygen therapy, *M* mechanical ventilation, *B* bronchodilators *C* corticosteroids

^*^ *P* < 0.05 compared to the changes in O group

### Survival analyses

Univariate and multivariate survival analyses were performed to identify the clinical parameters associated with mortality in CPFE (Table 6). After adjusting for potential confounders, PAH and a ≥ 5-point increase in CPI per year retained its statistical significance (Table 7). PAH patients had 10.29 times increased risk of death as compared to patients without PAH. The rate of increase in CPI by > 5 scores per year was associated with a 21.6 times higher risk of death.

**Table 6:** Univariate analysis: Relationship between clinical parameters and mortality in CPFE patients

Variable	*P*	Hazard ratio	95 % CI
BMI < 18 kg/m^2^	<0.01	5.64	2.68–11.89
Smoking	0.04	4.18	1–17.47
Diabetes	<0.01	5.19	2.51–10.75
Cardiovascular and cerebrovascular diseases	<0.01	4.68	1.65–13.28
Corpulmonale	<0.01	10.71	5.08–22.59
PAH	<0.01	27.68	8.35–91.81
Finger clubbing	<0.01	13.91	6.28–30.83
Increasing CEA level	<0.01	13.17	6.41–27.11
≥5-point increase of CPI per year	<0.01	45.03	15.19–133.52
≥5-score increase of HRCT per year	<0.01	4.63	2.47–8.71

*Abbreviations*: *CPFE* combined pulmonary fibrosis and emphysema, *BMI* body mass index, *PAH* pulmonary arterial hypertension, *CEA* carcinoembryonic antigen, *CPI* composite physiologic index, *HRCT* high-resolution computed tomography, *CI* confidence interval

**Table 7:** Multivariate analysis: Relationship between clinical parameters and mortality in CPFE patients

Variable	Estimated value	Standard error	Chi-square value	*P*	Hazard ratio	95 % CI
PAH	2.33	0.68	11.57	<0.01	10.29	2.69-39.42
≥5-point increase in CPI score per year	3.07	0.55	30.63	<0.01	21.60	7.28-64.16

*Abbreviations*: *CPFE* combined pulmonary fibrosis and emphysema, *PAH* pulmonary arterial hypertension, *CPI* composite physiologic index, *CI* confidence interval

## Discussion

Evidence suggests that a syndrome of CPFE, most frequently associated with IPF, deserves to be recognized as a separate clinical entity owing to the distinct clinical, functional, radiological, and pathological characteristics. In the present study, we compared the clinical characteristics, quantitative respiratory physiological index, radiographic scoring system, treatment and prognosis between patients with UIP plus emphysema (CPFE group) and IPF alone.

Although controversies exist in the published literature mainly regarding the prognosis of CPFE patients, the clinical characteristics of CPFE remain consistent with those described by Cottin et al. [9]. Most of the patients in the CPFE group in our study were males with a mean age between 65 and 70 years and had a smoking history which suggests tobacco smoking as a potential etiological factor for CPFE [7]. Perhaps the lower survival in CPFE group than IPF group might be partially explained by the difference in proportion of smokers in the two groups. Compared to patients with IPF, those with CPFE had a less frequent history of viral infection prior to the diagnosis, which suggests that viral infection may not be a risk factor for CPFE; the increasing trend in CEA with time; and basal crackles were more frequently observed on physical examination along with presence of finger clubbing. Surprisingly, over half of CPFE presented with wheeze, which was significantly more than that in patients with IPF alone. This suggests that airway spasm may be more common in CPFE, which might aggravate hypoxia concomitantly with hypercapnia. This pathophysiological change may cause extrapulmonary tissue damage and have an adverse effect on prognosis; therefore, CPFE may be considered as a more severe interstitial lung disease than IPF.

In this study, the chest HRCT and CPI scores were used for the first time to assess and monitor the course of CPFE. Simultaneous review of these two quantitative indices allowed for an objective evaluation of pulmonary structural and functional damage over the follow-up period. HRCT score and CPI tended to increase over time in both CPFE and IPF groups. However, the clinical course of CPFE varied from that of IPF. The increase in HRCT scores and CPI was more dramatic in the CPFE group, which suggested a correlation of these two indices with clinical deterioration of CPFE. These findings appear to justify the categorization of CPFE as a distinct syndrome that is characterized by a much worse prognosis than that of IPF. Survival analyses showed lower 5-year survival rate and poorer prognosis compared to IPF patients (43.42 % *vs*. 65.56 %; *P* < 0.05) and a higher mortality (39.47 % *vs*. 23.33 %; *P* < 0.05). However, evidence from recent studies that have compared the prognosis of CPFE with that of PF alone has largely been equivocal [6, 19, 20]. Cottin et al. reported a 5-year survival rate of 54.6 % among CPFE patients, which was higher than that observed in our study, but is still lower than that of the IPF group [9]. Mejla et al. reported one IPF group of 110 patients [6]. Of those, 31 (28 %) patients with CPFE had a higher mortality than that observed in IPF patients who did not have co-existing emphysema (median survival time: 25 *vs*. 34 months, *P* < 0.01). In contrast, others have reported either a better survival associated with CPFE as compared to that associated with IPF alone [12], or, no difference in mortality between the CPFE and IPF groups [20]. These conflicting results may be attributable to the selection of patients. Since consensus diagnostic criteria for CPFE have not yet been formally established, some studies included patients with interstitial lung diseases other than IPF, such as, nonspecific interstitial pneumonia (NSIP) which has a better prognosis than IPF. Additionally, the outcomes of CPFE may be influenced by the extent of PF associated with IPF, and which may take several clinical forms depending on the rate of progression to death [21].

Currently, there is a lack of consensus on the specific treatment strategy for CPFE. Studies evaluating therapeutic options for CPFE patients are still lacking. A few retrospective studies have reviewed the current treatment strategies for CPFE patients. Treatment has largely centered on the management of two separate components of CPFE: oral corticosteroids, immunosuppressive agents, N-acetylcysteine for PF and inhaled bronchodilators for emphysema, often supplemented with long-term oxygen therapy [7, 9]. Still, antifibrotic drugs used for patients with IPF may also be effective in those with pulmonary fibrosis (UIP) and emphysema. Owing to the retrospective nature of the present study, the effect of treatment on clinical outcomes of CPFE could not be evaluated. All 105 patients with IPF received oxygen therapy and corticosteroids treatment. Out of these, 15 patients who experienced acute exacerbation or respiratory failure during the disease course were administered noninvasive mechanical ventilatory support. Compared with CPFE group, bronchodilator treatment was not employed in IPF group. Therefore, the effect resulting from different treatments was likely to be very limited.

Univariate and multivariate survival analyses revealed PAH and a > 5-point increase in CPI score per year as predictors of mortality in CPFE. The risk of development of PH in CPFE patients was higher than those with IPF alone. Further, PH in CPFE patients was associated with an increased risk of death [6, 9, 22, 23]. Consistent with previous studies, PAH developed in nearly half of all patients in our CPFE group, and was a key determinant of mortality in this group (HR: 10.29, 95 % CI: 2.69–39.42). The development of PAH in CPFE may be attributed to the synergistic effect of PF and emphysema. In 2003, Wells et al. derived a composite physiologic index (CPI) from a group of IPF patients by fitting pulmonary function tests against disease extent as assessed on CT to correct the mortality risk assessment of IPF for the confounding effect of emphysema [15]. The CPI is simple to calculate based on the percent of predicted DLCO, FVC and FEV1, and is also a stronger predictor of mortality than the single index of pulmonary function [5, 13]. Our study suggests that a ≥ 5-point increase in CPI per year can be used as a predictor of mortality in CPFE patients (HR: 21.60, 95 % CI: 7.28–64.16), with the added advantage of being a non-invasive and easily obtainable physiological measure of prognosis.

Some limitations of our study are worth noting. Firstly, this study was a single-center retrospective study which precluded assessment of the effect of different therapeutic options on the outcomes of CPFE. Secondly, the possibility of selection bias cannot be ruled out since not all eligible subjects had complete radiographic and pulmonary function data available for analyses. Additionally, in the CPFE group, we only included patients who met the diagnostic criteria for IPF. CPFE represents a syndrome that has a characteristic clinical presentation, physiologic and radiographic findings, but also, possibly, pathological heterogeneity other than just IPF [9, 19]. Despite these limitations, the CPFE group in our study was reasonably well described, and characterized by typical findings.

## Conclusion

CPFE, a separate clinical syndrome that occurs predominantly in males and tobacco smokers, is characterized by distinct clinical, physiologic and radiographic features. The longitudinal trend of CPI and HRCT scores may mirror the clinical course of CPFE. CPFE has a more dismal prognosis compared to IPF alone. In this study, PAH and a 5-point increase in CPI per year were key predictors of mortality in patients with CPFE. Future prospective studies are needed to identify the optimal therapeutic approach to CPFE.

## Quick look

### Current knowledge

Combined pulmonary fibrosis and emphysema (CPFE) is increasingly acknowledged as a distinct syndrome with typical clinical, physiological and radiological characteristics. In clinical practice, CPFE cases are not uncommon. But the therapeutic and treatment options for patients with CPFE are limited.

### What this paper contributes to our knowledge

CPFE patients were predominantly male and smokers and exhibited distinct clinical, physiological and radiographic characteristics. They had a poorer prognosis than patients with idiopathic pulmonary fibrosis alone (IPF). Pulmonary arterial hypertension and ≥ 5-point increase in composite physiologic index (CPI) score per year were predictors of mortality in CPFE patients.

## Abbreviations

CPFE: Combined pulmonary fibrosis and emphysema
CPI: Composite physiologic index
CT: Computed tomography
HRCT: High-resolution computed tomography
ILD: Interstitial lung diseases
IPF: Idiopathic pulmonary fibrosis
PAH: Pulmonary arterial hypertension
PF: Pulmonary fibrosis
